# Biomechanical Evolution in the Neuropathic Diabetic Foot: A Scoping Review of Gait and Kinetic‐Kinematic‐Structural Adaptations in Diabetes

**DOI:** 10.1002/dmrr.70248

**Published:** 2026-11-04

**Authors:** G. Di Stasio, R. Colella, D. G. Armstrong, A. Bellingeri, A. De Mola, G. Montini, V. Scotti, A. De Silvestri, F. Campitiello

**Affiliations:** ^1^ Podos Logo Italia Naples Italy; ^2^ Phase I Clinical Trial Unit and Experimental Therapy IRCCS Fondazione San Matteo Pavia Italy; ^3^ Surgery and Neurological Surgery Keck School of Medicine of the University of Southern California (USC) Los Angeles California USA; ^4^ Wound Care Clinic IRCCS Fondazione San Matteo Pavia Italy; ^5^ ASST Crema Crema Italy; ^6^ University of Milan Milan Italy; ^7^ Research Library IRCCS Fondazione San Matteo Pavia Italy; ^8^ SSD Biostatistics and Clinical Trial Center IRCCS Fondazione San Matteo Pavia Italy; ^9^ General Oncology and Plastic Reconstructive Surgery Unit and Skin Ulcers and Diabetic Foot Unit Campania Region Reference Center Nostra Signora di Lourdes Hospital Naples Italy

**Keywords:** biomechanical phenomena, biomechanics, diabetic foot, equinus foot, lower limb, neuropathy, prevention, pronated foot, windlass mechanism

## Abstract

**Background:**

Gait alterations and biomechanical changes associated with diabetic peripheral polyneuropathy have been described in the literature, but the available evidence remains fragmented. It does not provide a comprehensive overview of how the reported biomechanical patterns differ across clinical stages of the Neuropathic Diabetic Foot (NDF). Mapping these patterns may support an improved understanding of the biomechanical mechanisms underlying increased ulceration risk.

**Aim:**

This scoping review aims to explore and synthesise the existing literature on changes in gait and foot biomechanics in people with diabetes in association with Diabetic Peripheral Neuropathy (DPN) and across its reported clinical stages.

**Methods:**

The scoping review was conducted in accordance with the JBI methodology for scoping reviews and in line with the Preferred Reporting Items for Systematic Reviews and Meta‐Analyses extension for Scoping Reviews (PRISMA‐ScR). The population included adults with diabetes and peripheral neuropathy, without active foot ulceration, who were managed in community or outpatient settings. The concept of interest is gait and foot‐biomechanical alterations associated with diabetic neuropathy in clinical and biomechanical assessment contexts. A comprehensive search was conducted in PubMed, EMBASE, CINAHL, PEDro, and the Cochrane Library, complemented by grey literature sources including the Trip Medical Database, NICE, and the Guidelines International Network (GIN). Studies published within the last 11 years were considered (2014/12–2026/04). Data were charted and synthesised using a descriptive and narrative approach.

**Results:**

From 2044 initial records, 44 sources met the eligibility criteria. An updated search conducted in April 2026 identified 6 additional eligible sources, resulting in 50 included sources. The evidence mapped across three principal themes: gait and lower‐limb kinematic alterations, changes in plantar loading and joint kinetics, and structural or functional changes of the foot. No included study directly evaluated whether the identified biomechanical alterations predicted the subsequent development of Charcot neuro‐osteoarthropathy.

**Conclusions:**

To our knowledge, this is the first scoping review to map structural, kinematic, kinetic, and plantar‐loading findings within an integrated biomechanical framework of the neuropathic diabetic foot. Because the evidence was predominantly cross‐sectional, the proposed relationships should be interpreted as a conceptual synthesis rather than a demonstrated longitudinal progression. These findings highlight the need for longitudinal studies to investigate the temporal evolution of biomechanical alterations in the neuropathic diabetic foot.

## 1 Introduction

Foot‐related complications represent one of the leading causes of morbidity among individuals with diabetes, particularly in the presence of peripheral neuropathy [1]. The lifetime risk of developing a diabetes‐related foot ulcer has been estimated to range between 19% and 34%, with high recurrence rates following initial healing [2]. According to the 2023 International Working Group on the Diabetic Foot (IWGDF) guidelines, effective prevention relies on early identification of at‐risk feet, regular foot assessments, patient and healthcare professional education, appropriate footwear, and timely management of risk factors. Therefore, preventing foot ulceration remains a fundamental clinical priority [2].

Lower‐limb complications constitute a pervasive, debilitating, and economically burdensome clinical manifestation in individuals affected by NDF [1, 3, 4]. Within this pathological cascade, plantar overload syndromes and repetitive mechanical microtrauma represent the principal proximal precursors of diabetic foot ulceration (DFU) and the subsequent risk of amputation [4]. Historically, approximately 50% of major and minor diabetes‐related amputations in the United States have been directly attributed to the use of inappropriate footwear and/or inadequate plantar pressure offloading strategies [1]. In most cases, the onset of these lesions results from cumulative spatiotemporal gait deficits, unsuitable footwear choices, or acute traumatic insults that remain entirely unnoticed due to the loss of protective sensation [1, 4].

Current epidemiological evidence underscores the global magnitude of this healthcare challenge. Diabetes‐related lower‐limb complications, together with ulcerations of vascular and rheumatological origin, impose an increasing burden of disability that negatively affects patients' quality of life and generates unsustainable healthcare costs [1, 5]. Owing to their high prevalence, poor prognostic implications, and high recurrence rates, chronic lower‐extremity ulcerations have emerged as a significant public health issue, necessitating a clear transition from purely reactive treatment approaches towards structured primary prevention programs [5]. Within this therapeutic framework, implementing mechanical protection strategies assumes a crucial role [1].

The development and progression of NDF not only substantially alter gait biomechanics but also actively exacerbate any pre‐existing lower‐limb biomechanical dysfunctions that preceded the clinical diagnosis, thereby amplifying the cumulative pathological burden [1, 4]. This clinical scenario underscores the need for podiatric medicine to continue evolving towards Evidence‐Based Practice models, thereby redefining its autonomy and central role within the multidisciplinary team [2]. The management of the complex diabetic foot cannot be separated from the patient's systemic pathophysiological condition; consequently, podiatric autonomy must be integrated with validated screening protocols that identify and map clinical and biomechanical risk factors before tissue damage occurs [2].

Diabetic foot syndrome should be conceptualised as a complex and inherently multifactorial condition in which peripheral neuropathy, macrovascular and microvascular disease, progressive structural deformities, and altered plantar loading patterns interact synergistically to accelerate soft‐tissue breakdown [1, 4]. The pathophysiological cascade triggered by DPN extends far beyond the simple loss of protective sensation. It includes autonomic nervous system dysfunction, which leads to anhidrosis and consequent skin fragility, as well as motor neuropathy, which causes intrinsic foot muscle atrophy, muscular kinetic imbalance, secondary digital deformities such as claw toes and hammer toes. Simultaneously, non‐enzymatic glycation of collagen fibres increases the structural stiffness of key soft tissues, including the Achilles tendon and plantar fascia, significantly restricting overall joint mobility [4].

Despite ongoing technological advances in screening systems and orthotic offloading strategies, a substantial proportion of ulcerations remains preventable. This persistent clinical gap underscores the need to map the precise biomechanical mechanisms that govern risk progression through a scoping review of the existing literature [2]. Numerous studies have examined the effects of DPN on gait, consistently demonstrating alterations in spatiotemporal parameters, multisegmental joint kinematics and kinetics, and both vertical and shear plantar pressure distributions [1, 3].

Current international guidelines on therapeutic footwear and orthotic interventions provide high‐level evidence and strong clinical recommendations primarily for patients already classified as high risk or with a history of ulceration or Charcot neuro‐osteoarthropathy [1, 2, 3]. However, the literature reveals a marked scarcity of validated biomechanical models to guide and standardise primary prevention strategies for patients who have not yet developed open lesions [4]. Extending these conservative and personalised interventions to patient cohorts in the early stages of disease represents a cornerstone of primary prevention. Therefore, this scoping review aims to map and critically evaluate the literature on screening protocols, biomechanical prevention strategies, and podiatric treatments to synthesise a novel clinical model that bridges the gap between advanced laboratory research and proactive preventive care in everyday outpatient practice [2, 5].

## 2 Methods

We conducted this scoping review following the JBI methodology for scoping reviews [6]. We used the PRISMA‐ScR Checklist for reporting [7]. The scoping review protocol was registered in the OSF registry [8].

### 2.1 Research Team

To support robust, clinically relevant findings, the research team included authors with expertise in evidence synthesis, quantitative research methodology, biomechanics, statistics, and bibliographic research.

### 2.2 Review Questions

1. What biomechanical parameters are used to characterise the diabetic foot in the different stages of neuropathy?

Sub‐questions include:

2. How has the evolution of gait and foot mechanics in the neuropathic diabetic foot been described in the literature?
3. Which assessment methods and measurement tools are used to evaluate these biomechanical changes?
4. Which structural, kinematic, kinetic, and plantar loading alterations are reported?
5. How are neuropathy stage, deformity progression, and ulceration risk conceptualised in the included studies?
6. Which study designs and methodological approaches have been used to investigate this topic?
7. What knowledge gaps emerge from the available literature?

### 2.3 Eligibility Criteria

Studies were eligible for inclusion if they met the following Population, Concept and Context (PCC) criteria.

- *Population.* Patients with diabetes and diabetic neuropathy; patients managed in community or outpatient settings.
- *Concept.* The concept of interest is the biomechanics of gait and foot function in adults with diabetic peripheral neuropathy. This includes structural, kinematic, kinetic, plantar loading, and functional alterations, as well as the methods used to assess these parameters and their reported relationship with deformity progression or ulceration risk.
- *Context.* This review considered studies conducted in ambulatory settings and laboratory‐based gait analyses involving non‐hospitalised individuals living in the community.
- *Types of evidence sources.* This scoping review included all study designs and publication types. We included studies in all languages with an English abstract. No geographical restrictions were applied. We considered studies from 12/2014 to 04/2026.

Exclusion criteria included hospitalisation and active DFUs.

Peripheral Arterial Disease (PAD) conditions may influence tissue integrity and the interpretation of biomechanical findings. Therefore, studies including participants with PAD were considered only if PAD was reported as a co‐existing clinical characteristic within a primary neuropathic population [2].

We considered foot posture and structural alignment when reported and did not use any foot‐posture category as an eligibility criterion. We retained abnormal pronation as a prespecified exploratory question in accordance with the registered protocol [8]; this focus neither restricted inclusion to participants with pronated feet nor implied a causal relationship with Charcot neuro‐osteoarthropathy.

To ensure feasibility, we limited the search to the last decade; however, we included seminal studies identified through citation tracking. Several important papers on kinetic outcomes were published for the first time [9, 10, 11, 12, 13, 14, 15, 16], and we evaluated secondary literature on kinematic and structural outcomes, enriching all three outcome domains with higher‐quality papers [17, 18, 19, 20].

### 2.4 Search Strategy

We conducted an initial limited search of MEDLINE via the PubMed interface to identify index terms used to describe articles on the topic. We then used these terms to develop a comprehensive MEDLINE search strategy. We adapted this strategy, including all identified keywords and index terms, for PubMed, EMBASE, CINAHL, PEDro, and Cochrane up to April 2026, capturing clinical trials, observational studies, reviews, and guidelines.

An experienced librarian conducted the searches at the scientific library of the San Matteo Polyclinic in Pavia. For grey literature, additional bibliographic sources consulted included the Trip Medical Database, NICE, and the GIN. Additionally, we contacted and discussed the topic with experts in the field to identify additional sources (see Supporting Information S1: File S1). The authors thank the Italian Association for Tissue Repair and Regeneration (AIRRT) for methodological support.

### 2.5 Study Selection

An expert librarian deduplicated all records using Rayyan. We performed additional manual duplicate checks before initiating the review process. Two pairs of reviewers independently screened titles and abstracts retrieved through the search strategy, as well as those identified from additional sources, using Rayyan to identify studies meeting the inclusion criteria.

Both reviewers retrieved the full texts of potentially eligible studies and independently assessed them for eligibility. Reviewers resolved disagreements about including specific studies through discussion or by consulting a third reviewer (though this did not occur). We recorded excluded studies in a PRISMA flow diagram, along with their corresponding reasons for exclusion.

### 2.6 Data Extraction and Data Synthesis

Two pairs of reviewers independently conducted data extraction using a structured extraction form. The extraction protocol was piloted on the first five included studies to ensure process consistency; necessary modifications to the data extraction form were made and documented.

Data extracted from each article included the following domains:

- Study details (e.g., name of first author, year of publication, country, funding source, setting);
- Description of the study population and participant demographics, including baseline characteristics;
- Study design and methodology;
- Population, concept, context;
- Information on the intervention(s);
- Details of any comparison group(s).

The synthesis of the results was narrative. In particular, the studies were grouped according to three macrodomains (Kinematic, Kinetic, and Structural), setting types, populations, study designs, and diagnostic criteria for NDF. We summarised biomechanical parameters and their measurement tools in Synthesis Without Meta‐analysis tables, and reported methodological design classifications to contextualise evidence maturity. To understand how measurement‐method resolution influences the interpretation of biomechanical risk and to identify knowledge gaps, we classified biomechanical parameters into three levels of granularity, as reported in Supporting Information S2: File S2.

## 3 Results

As shown in the PRISMA 2020 flow diagram (Figure 1), the initial literature search yielded 2044 records. After removing duplicates, 1263 studies were screened, of which 44 articles were initially included. In April 2026, an updated literature search identified 336 additional records; after screening, 6 more articles were included. In total, 50 records were included in this review [9, 10, 11, 12, 13, 14, 15, 16, 17, 18, 19, 20, 21, 22, 23, 24, 25, 26, 27, 28, 29, 30, 31, 32, 33, 34, 35, 36, 37, 38, 39, 40, 41, 42, 43, 44, 45, 46, 47, 48, 49, 50, 51, 52, 53, 54, 55, 56, 57, 58].

**FIGURE 1:**
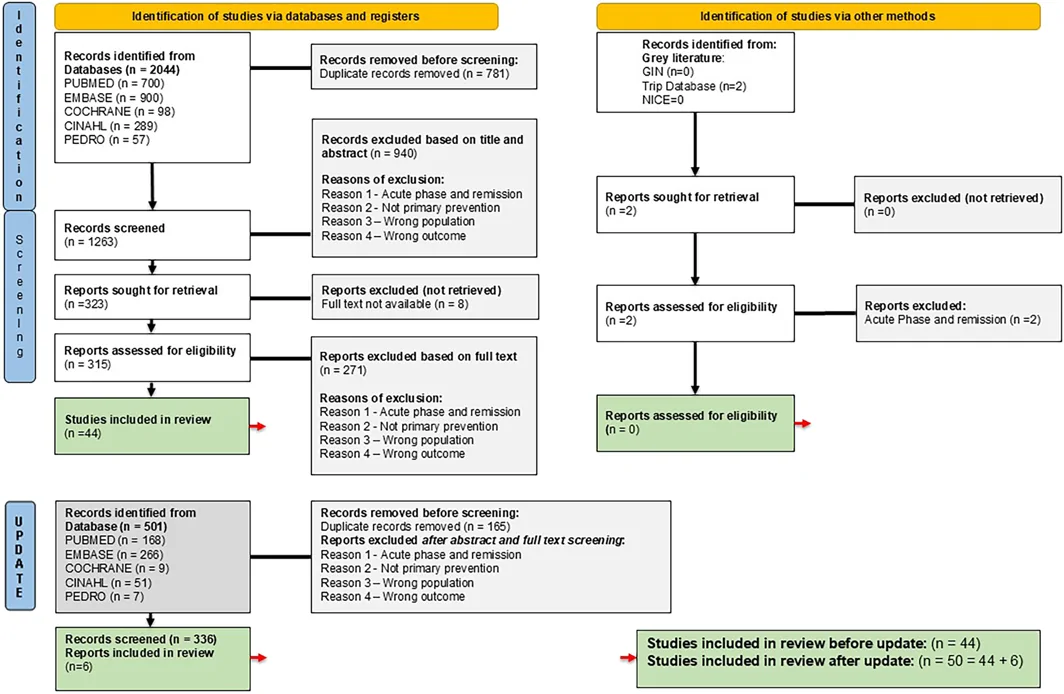
PRISMA 2020 flow diagram.

### 3.1 Characteristics of Included Studies

Table 1 synthesises the main characteristics of the studies. To ensure transparent reporting, Supporting Information S3: File S3 shows the complete extracted data for each included study.

**TABLE 1:** Characteristics of the included sources.

Years of publication	Number of papers (%)
December 2014–December 2019 (61 months)	22 (44.0%)
January 2020–December 2024 (60 months)	22 (44.0%)
January 2025–April 2026 (16 months)	6 (12.0%)
Total sources included: December 2014–April 2026 (137 months)	50 (100.0%)
Paper design	
Primary papers	45 (90.0%)
Cross‐sectional papers	32 (64.0%)
Case‐control papers	7 (14.0%)
Experimental papers	6 (12.0%)
Secondary papers	5 (10.0%)
Systematic reviews and systematic surveys (including 2 meta‐analyses)	4 (8.0%)
Narrative reviews	1 (2.0%)
Total papers included	50 (100.0%)
Biomechanical outcome domains	
Kinematic outcomes	37 (74.0%)
Papers only in the kinematic domain	0 (0%)
Kinetic outcomes	41 (82.0%)
Papers only in the kinetic domain	1 (2.0%)
Structural outcomes	40 (80.0%)
Papers only in the structural domain	2 (4.0%)
Overlapping outcome domains	
Kinematic and structural outcomes	7 (14.0%)
Kinetic and structural outcomes	10 (20.0%)
Kinematic and kinetic outcomes	9 (18.0%)
Kinematic, kinetic, and structural outcomes	21 (42.0%)

*Note:* Outcome‐domain categories were not mutually exclusive because individual sources could report findings in more than one biomechanical domain. We calculated percentages using all 50 included sources as the denominator. The 45 primary studies and 5 secondary sources were mutually exclusive categories. Kinematic outcomes are ‘aprospulsive gait/sagittal plane deficit/swinging gait in the frontal anatomical plane OR Risk of falling/tripping OR flexed knees OR Positive/negative work of the midfoot’. Kinetic outcomes are ‘Ankle moment OR Maximum ankle power generation OR Positive/negative ankle work OR Metatarsal moment OR Maximum metatarsal power generation/Plantar loading test’. Structural outcomes are ‘Equinus foot OR Pronate foot OR Functionally limited hallux/Hallux rigidus OR Windlass/reverse windlass mechanism OR Extra rotation of the lower limbs/Pelvic misalignment/Abducted feet OR Hallux valgus’.

The 50 included sources comprised 45 primary studies and 5 secondary sources. Among the primary studies, 32 used cross‐sectional designs, 7 used case‐control designs, and 6 were experimental. We identified no longitudinal or prospective cohort studies investigating the temporal progression of biomechanical alterations during DPN evolution. Secondary sources included three systematic reviews [17, 18, 19], two meta‐analyses [18, 19], one systematic survey [20], and one narrative review [12].

Study populations primarily included adults with diabetes mellitus, with a substantial proportion diagnosed with DPN. Several studies used comparative designs that included diabetic patients without neuropathy and/or healthy controls, enabling indirect comparisons across disease stages [21, 25, 35, 49]. Sample sizes varied considerably across studies, generally ranging from approximately 10–20 participants in smaller experimental studies to approximately 30–60 participants in moderate‐sized observational studies.

Most studies were conducted in controlled biomechanical laboratory settings using instrumented gait analysis systems. Measurement techniques included three‐dimensional motion capture systems (e.g., Vicon), plantar pressure measurement systems (e.g., Pedar), force platforms, wearable inertial measurement units, and electromyography [14, 23, 26]. A limited number of studies have evaluated biomechanical parameters in real‐world or community‐based settings [20, 58].

### 3.2 Biomechanical Domains

The extracted data were categorised into three predefined biomechanical outcome domains: kinematic, kinetic, and structural. Several studies assessed more than one biomechanical domain in the same participants.

#### 3.2.1 Kinematic Outcomes

Kinematic outcomes were reported in 37 included sources and primarily concerned spatiotemporal gait parameters and lower‐limb joint motion. Studies comparing participants with DPN with non‐diabetic controls or participants with diabetes without neuropathy reported lower walking speed, shorter step or stride length, greater step width, longer double‐support time, and greater gait variability [10, 17, 19, 21, 24, 35, 41, 42, 49, 53]. Additional reported findings included differences in hip and knee flexion, ankle and first metatarsophalangeal joint range of motion, step‐progression angle, foot elevation at toe‐off, and subtalar or foot‐segment motion [19, 25, 34, 41, 49, 53].

Kinematic analyses reported reduced ankle range of motion, particularly in the sagittal plane, including lower dorsiflexion during stance and lower plantarflexion during late stance or push‐off in some DPN groups [10, 25, 35, 49].

A systematic review and meta‐analysis by Hazari et al. reported a between‐group difference in knee‐flexion range [19]. Yamasaki et al. reported lower walking ranges of motion at the knee, ankle, and first metatarsophalangeal joint in participants with a history of diabetic foot ulcer than in participants with diabetes without DPN [53].

#### 3.2.2 Kinetic Outcomes

Kinetic outcomes were reported in 41 included sources and primarily concerned plantar pressure distribution, plantar shear, ground reaction forces, joint moments, power, and mechanical work. In a comparison of participants with DPN, diabetes without neuropathy, and non‐diabetic controls, the DPN group had higher global peak shear, shear–time integral, and pressure–time integral [38].

A consistent finding across studies was increased peak plantar pressure in the forefoot region, often under the central metatarsal heads [11, 13, 18, 19, 21, 23, 24, 26, 27, 28, 29, 31, 35, 36, 37, 38, 41, 43, 44, 45, 48, 50, 51, 54, 56, 57, 58].

Ground reaction forces changed, including loading rates and the temporal characteristics of the stance. Reduced propulsive forces during push‐off were frequently reported [9, 10, 15, 49], with impaired forward progression [10, 15, 38].

Joint kinetic analyses demonstrated reduced ankle power generation and altered ankle moments, reporting decreased mechanical efficiency and reduced energy transfer during gait [14, 46]. These findings reflect a transition towards ‘decreased net mechanical work’ of the foot with an ‘energy‐neutral foot’, in which the balance between energy absorption and generation approaches equilibrium [9]. At the midtarsal level, altered energy absorption patterns and increased negative work have been reported, contributing to cumulative mechanical stress within the foot structures [16, 49]. These altered foot mechanics affect the longitudinal arch and windlass mechanisms [22].

Across neuropathy‐severity groups, studies reported plantar‐pressure differences in the midfoot, heel, and medial forefoot although the direction of these differences was not uniform across regions. The included studies did not show a uniform increase across all plantar‐loading variables. In the largest diabetic‐versus‐non‐diabetic comparison, participants with diabetes had longer contact time and higher maximum force, pressure–time integral, and force–time integral. Joint kinetic analyses have reported differences in ankle, rearfoot, midfoot, and proximal‐joint mechanics [9, 14, 16, 46, 49, 52].

DiLiberto et al. reported greater negative peak power and lower positive peak power at the midfoot and rearfoot in participants with DPN than in controls [16].

Deschamps et al. reported lower midfoot peak power generation and positive work and a higher negative‐work ratio in DPN, whereas several joint‐moment comparisons were not statistically significant [9].

During single‐limb heel rise, ankle plantarflexion power was lower in participants with DPN and medial‐column deformity than in controls [46].

During speed‐controlled walking, the DPN group showed greater ankle power absorption during late stance and a delayed and shorter power‐generation phase [49].

Differences in external ankle moment arm, effective mechanical advantage, and ankle joint moment were also reported among the DPN, diabetes without neuropathy, and control groups [52].

Studies including participants with DPN and PAD reported between‐group differences in peak pressure, pressure–time integral, contact area, and centre‐of‐pressure variables [24, 50].

#### 3.2.3 Structural Outcomes

Structural outcomes were examined in 40 included sources and described morphological and functional characteristics of the foot and lower limb. Reported findings included limited ankle dorsiflexion or equinus [18, 27, 31, 38, 53], hypermobility of the first ray, and alterations in the windlass mechanism [27, 38, 39], and deformities including hallux valgus, hallux rigidus or limited first‐metatarsophalangeal joint motion, lesser‐toe deformity, altered forefoot alignment, medial‐column deformity and changes in arch structure [29, 30, 33, 34, 43, 46].

Other reported structural measures included Achilles tendon and plantar fascia thickness and stiffness, intrinsic foot muscle and fat volumes, tendon‐to‐volume ratios, medial column alignment, plantarflexor torque, and reduced structural stability of the foot [9, 22, 30, 34]. Studies also reported differences in foot‐progression angle and distal and proximal lower‐limb joint motion [10, 19, 41, 49, 53]. However, the included studies did not establish a temporal progression from equinus to pronation or subsequent structural deformity.

#### 3.2.4 Integration Across Biomechanical Domains

Although outcomes were categorised into distinct domains, many studies reported overlapping findings across kinematic, kinetic, and structural domains. For example, weight‐bearing ankle dorsiflexion was associated with higher barefoot forefoot peak pressure and pressure–time integral, as well as changes in propulsion mechanics [14, 38, 40]. Hallux valgus and hallux rigidus were associated with higher medial‐forefoot peak pressure and with increased forefoot loading [30, 31, 43], while fifth‐toe deformity was associated with differences in regional plantar loading [44]. Other studies concurrently assessed foot‐segment motion and joint moments, power, or mechanical work [9, 16, 46, 49].

No included study directly evaluated whether pronated foot posture, reduced midfoot power generation, or other identified biomechanical alterations predicted the subsequent development of Charcot neuro‐osteoarthropathy.

## 4 Discussion

This scoping review provides a comprehensive mapping of the current evidence on biomechanical alterations in NDF, highlighting three interrelated domains: gait adaptations, altered plantar load distribution, and structural foot deformities. Across the included studies, DPN was consistently associated with measurable alterations in spatiotemporal gait parameters (kinematic outcomes), impaired load modulation (kinetic outcomes), and morphological (structural) changes of the foot [17, 31, 42, 46]. The shift towards a diabetic equinus foot state may induce subtalar and midfoot collapse/pronation, earlier heel rise, increased knee flexion, and an external foot progression angle (‘toe‐out’) [59, 60].

However, the evidence is predominantly derived from cross‐sectional studies, limiting the ability to determine temporal sequences or causal pathways, particularly regarding disease progression and ageing [26, 31]. A key finding of this review is the fragmentation of the literature, with most studies focussing on isolated biomechanical variables rather than integrated models of disease progression.

Interpreting gait alterations in DPN requires careful consideration. These changes may reflect compensatory strategies to maintain stability in the presence of sensory and proprioceptive deficits, rather than direct indicators of pathological deterioration. This distinction is clinically relevant because it challenges the assumption that all biomechanical deviations in DPN should be interpreted as risk factors. Some adaptations may serve a protective or stabilising function. From a clinical perspective, this raises the question of whether interventions should aim to correct these deviations or support them. For instance, therapeutic approaches may need to be designed to facilitate functional compensation rather than suppress it.

Reduced ankle power generation and altered joint kinetics may be suggestive of impaired propulsion and decreased mechanical efficiency [14, 23]. These changes are commonly interpreted as contributing to increased mechanical stress on plantar tissues and foot joints. However, the causal relationship between plantar pressure abnormalities and ulcer formation remains insufficiently established. Most available studies report statistical associations rather than longitudinal progression towards clinically relevant outcomes [31, 46]. Furthermore, it remains unclear to what extent plantar pressure abnormalities directly contribute to DFU development, as factors such as loading duration, cumulative stress exposure, and tissue tolerance are also likely to play important roles.

PAD may represent a coexisting factor when interpreting plantar‐loading findings in people with diabetes and peripheral neuropathy. The included cross‐sectional studies reported differences in regional plantar pressure, pressure–time integral, contact area, and centre‐of‐pressure variables among participants with DPN, PAD, or both conditions [24, 50]. From a pathophysiological perspective, PAD‐related ischaemia may reduce tissue tolerance and the capacity to repair repetitive microinjury, thereby magnifying the effects of mechanical loading [2]. However, the included studies did not directly evaluate tissue hypoxia, tissue repair capacity, or plantar fat pad changes as mechanisms underlying the observed loading patterns.

Structural alterations are frequently attributed to intrinsic muscle weakness, joint stiffness, and cumulative mechanical stress [9, 22]. Within a conceptual framework, these changes may represent a later stage of biomechanical dysfunction, reflecting the long‐term consequences of altered loading patterns and motor control. However, the absence of longitudinal data prevents confirmation of this temporal sequence. It remains unclear whether structural changes are a consequence of altered biomechanics or a contributing factor to their development.

Kinetic and structural findings suggest that individuals with polyneuropathy exhibit reduced sagittal angular velocity across the foot segments, resulting in decreased midtarsal power generation [9]. This reduction diminishes the foot's contribution to forward propulsion, as the foot tends to reach an ‘energy‐neutral’ state in which net mechanical work is near zero or slightly negative (a near balance between positive and negative work). This energy‐neutral state has been described as a progressive loss of the foot's normal energy‐generating function. Key findings regarding this condition could include:

1. Mechanical Consequences: The shift towards an energy‐neutral foot may increase mechanical demands on passive structures.
2. Pathophysiology: The underlying mechanisms may include a combination of limited joint mobility, increased stiffness of the Achilles tendon and plantar fascia, and distal muscle weakness.
3. Structural Deformities: This phenomenon contributes to cumulative tissue stress, potentially leading to foot deformities and progressive joint degeneration.
4. Compensatory Strategies: Altered kinematics at the tarsometatarsal joint may represent compensatory strategies in response to impaired distal function.

This condition appears to occur in individuals with NDF who exhibit reduced midtarsal power, often characterised by a decreased ability of the foot to function as a rigid lever during gait [9]. The findings of this review suggest that biomechanical alterations in DPN should not be interpreted as isolated phenomena but as potentially interconnected components of a complex system. The coexistence of kinematic, kinetic, and structural findings provides a hypothesis‐generating biomechanical framework that could be relevant to Charcot neuro‐osteoarthropathy; however, no included study directly tested this relationship.

Accordingly, the potential relationship between foot posture, altered midfoot energetics, and the subsequent development of Charcot neuro‐osteoarthropathy remains an evidence gap.

One of the main contributions of this review is identifying the lack of integrated biomechanical models in the literature. Based on the synthesis of the available evidence, a conceptual framework can be proposed in which sensorimotor impairment, gait adaptations, altered load distribution, and structural changes may interact. However, this framework should be interpreted with caution; it represents a theoretical integration of fragmented findings rather than a validated progression model [26, 31].

Kinematic analyses revealed reduced ankle ROM, particularly in the sagittal plane, with decreased dorsiflexion during stance and reduced plantarflexion during push‐off [10, 25, 35]. These alterations suggest impaired propulsion and reduced efficiency during terminal stance, indicative of equinus foot and windlass mechanism deficits. Combining data on step‐distance factors, joint ROM limitations, and joint function limitations suggests that as DPN progresses, gait transitions to a ‘shuffle walk’ characterised by short step lengths, wide step widths, and reduced joint ROM below the hip. Although diabetic shuffle walking and an enlarged step width lead to a more stable gait and reduce the risk of falling, the accompanying increase in pressure and shear forces in the plantar region promote callus formation and can lead to foot ulceration [53].

Figure 2 presents a hypothetical model/paradigm of the changes in a diabetic patient's gait pattern associated with worsening diabetic polyneuropathy, including biomechanical and postural changes, integrating kinematic, kinetic, and structural findings mapped in this review.

**FIGURE 2:**
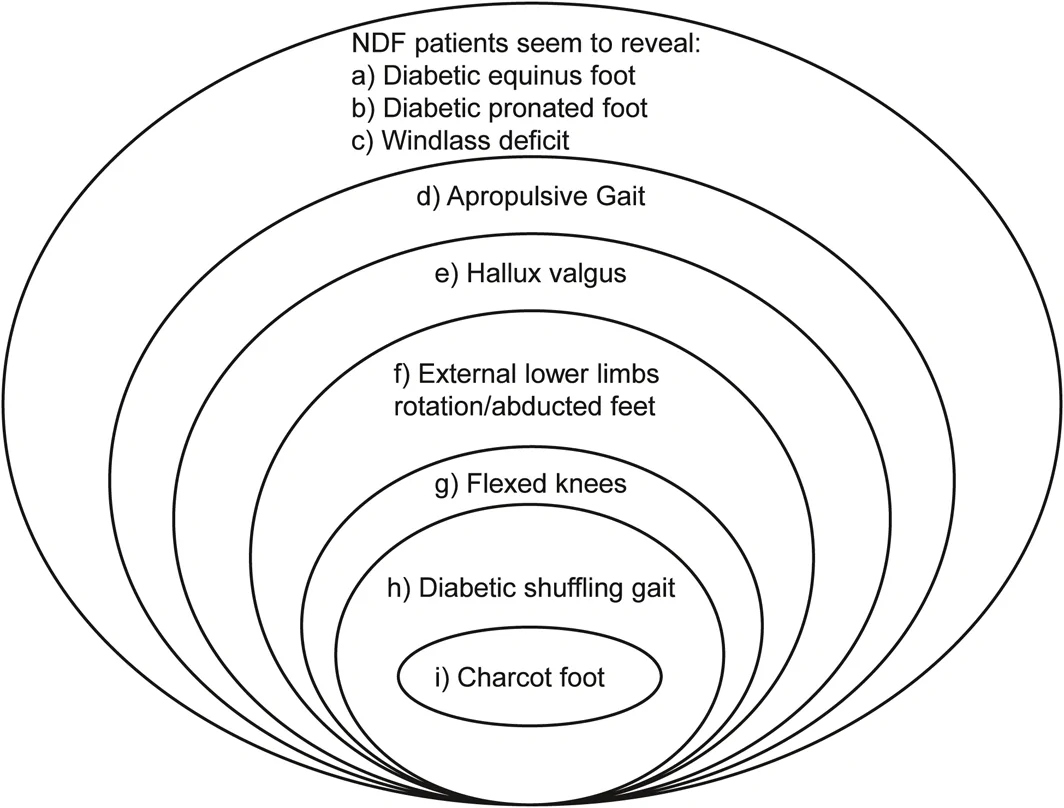
The Venn diagram shows all patients with diagnosed diabetic neuropathy; moving from one ellipse to the next, from the outside to the inside, reflects an hypothetical increasing severity of the condition. This idealised representation illustrates that, as diabetic neuropathy worsens, new postural and biomechanical deficits seem to emerge, resulting from the aggravation of the equinus diabetic foot. The innermost circle depicts Charcot foot, provided that it can be demonstrated that charcot foot also has a biomechanical nature. These poly‐NDF compensation subsets are hypothesised although they have yet to be proven. This figure illustrates the transition from ‘normal’ to ‘pathological’ foot biomechanics in people with diabetes, before and after the onset of diabetic peripheral neuropathy and a diagnosis of DF: a conceptual synthesis across largely cross‐sectional evidence, not a demonstrated longitudinal natural history.

Figure 3 presents a hypothesis‐generating conceptual framework integrating the sensorimotor, kinematic, kinetic, loading, and structural findings mapped in this review. Bidirectional arrows represent potentially interacting relationships rather than an established temporal or causal sequence. The figure should therefore be interpreted as a conceptual synthesis of predominantly cross‐sectional evidence and not as a validated model of the longitudinal progression of the neuropathic diabetic foot.

**FIGURE 3:**
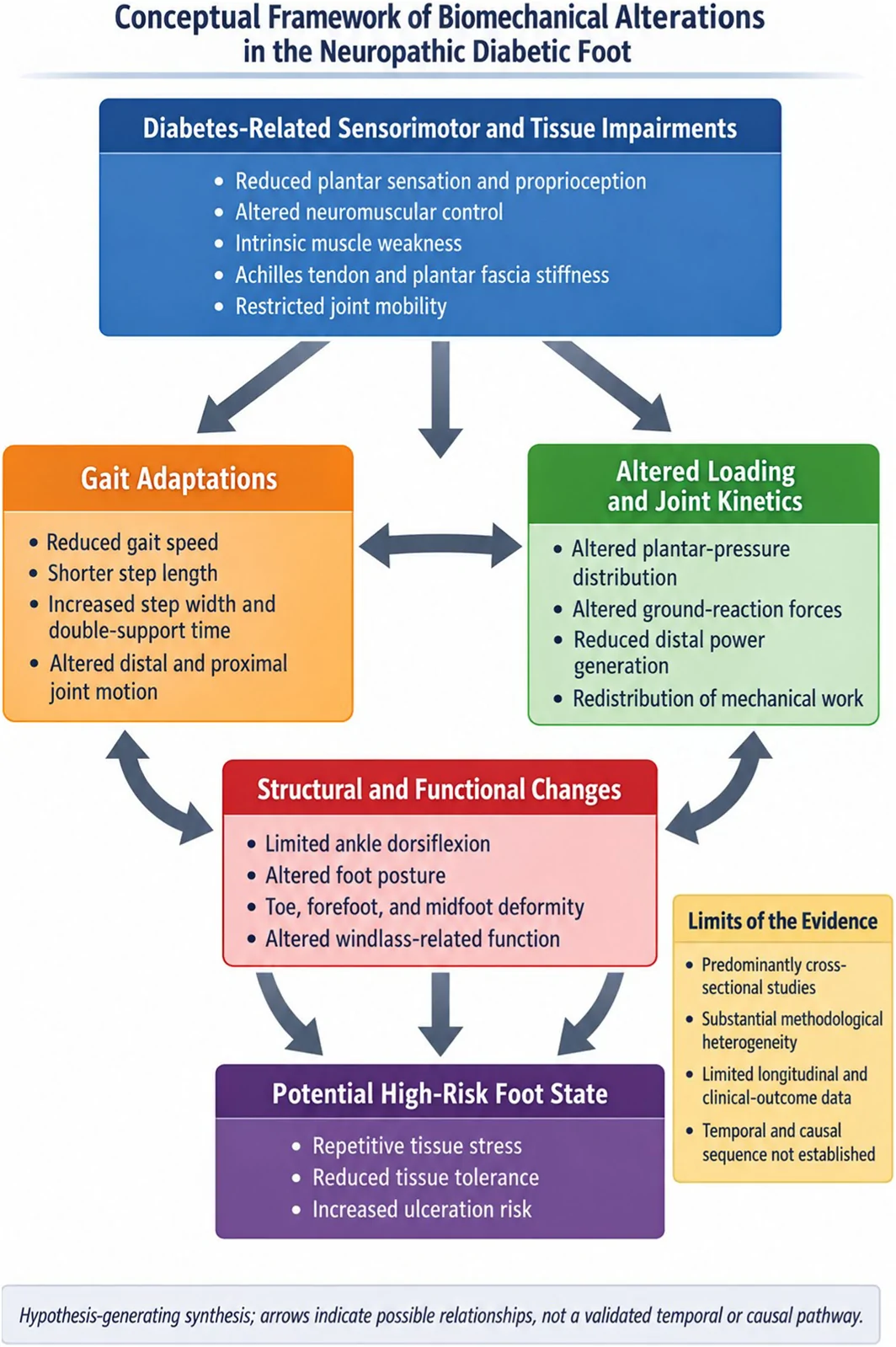
Hypothesis‐generating conceptual framework of biomechanical alterations in the neuropathic diabetic foot. Diabetes‐related sensory, neuromuscular, muscular, connective‐tissue, and joint impairments may interact with gait adaptations, altered loading and joint kinetics, and structural or functional foot changes. Bidirectional arrows indicate possible reciprocal relationships among these domains. Repetitive tissue stress, together with reduced tissue tolerance, may contribute to a high‐risk foot state. The framework is an author‐derived synthesis of predominantly cross‐sectional evidence and does not represent a validated temporal or causal pathway.

### 4.1 Clinical Implications

Despite these limitations, the findings suggest that early biomechanical alterations in DPN may be detectable through gait analysis and plantar pressure assessment [40, 42]. From a clinical perspective, this supports the potential role of early screening strategies focused on functional biomechanical parameters. However, caution is required when translating these findings into predictive or prognostic models given the lack of longitudinal validation.

Although this scoping review does not allow definitive conclusions regarding the effectiveness of specific interventions, it provides a comprehensive overview of biomechanical alterations in individuals with DPN. The findings highlight the importance of considering biomechanical factors in clinical assessment and management. In particular, integrating gait analysis, plantar pressure assessment, and biomechanical functional testing [61] may help identify at‐risk individuals early.

### 4.2 Strengths and Limitations

First, the evidence base is predominantly cross‐sectional, limiting the ability to determine whether observed biomechanical alterations reflect progressive changes over time or static differences between groups, thereby restricting causal inference and the verification of temporal relationships. Second, there is substantial heterogeneity in study methodologies, relating to differences in measurement techniques, gait analysis systems, plantar pressure devices, outcome definitions, and data reporting, which limits comparability across studies.

The prespecified exploratory focus on pronation should not be interpreted as implying that it is the only clinically relevant foot posture in DPN. This review was not designed to compare foot‐posture categories. Although one cross‐sectional study found an association between pronated foot posture and previous forefoot ulceration [39] and it seems that equinus deformity induces abnormal pronation of the foot [59], no included study established abnormal pronation as a predictor or causal precursor of Charcot neuro‐osteoarthropathy.

Another serious limitation is that most studies relied on biomechanical, non‐diagnostic tests. The lack of information on the sensitivity and specificity of these tests limits their reliability/validity/measurement error; flaws in these metrics can lead to random and systematic errors in the reported results [61].

### 4.3 Future Research Directions

Future research should prioritise longitudinal study designs to investigate the progression of biomechanical alterations in DPN. Integrated analytical approaches that combine kinematic, kinetic, and structural data within unified models are also needed. Standardisation of measurement techniques and outcome reporting would significantly improve comparability across studies. Future studies should establish clear links between biomechanical alterations and clinically relevant endpoints to enhance the translational value of research in this field.

Recent evidence indicates that individuals with diabetes and peripheral neuropathy may exhibit reduced power generation and altered mechanical work at the midfoot and other foot joints [9, 16]. In particular, reduced midfoot power generation and a greater contribution of negative work have been reported, indicating an altered balance between energy absorption and generation during gait [9, 16]. These kinetic findings have been discussed within the broader concept of an ‘energy‐neutral foot.’ However, any proposed relationship among reduced midfoot power generation, increased mechanical demands on passive structures, foot pronation, and Charcot neuro‐osteoarthropathy therefore represents a theoretical interpretation of the available findings rather than a demonstrated association. These interpretations remain preliminary and require further investigation.

Nevertheless, the absence of direct evidence does not invalidate the protocol‐defined exploratory question; rather, it identifies the relationship between abnormal pronation and Charcot neuro‐osteoarthropathy as a specific evidence gap.

## 5 Conclusion

The evidence synthesised in this review suggests that the neuropathic diabetic foot is characterised by a complex interplay among gait adaptations, altered plantar load distribution, and progressive structural changes in the foot, supporting the concept of an integrated biomechanical dysfunction. Several studies have identified foot pronation (first ray hypermobility, windlass deficit, longitudinal arch reduction, altered forefoot alignment, medial‐column deformity) as a common feature in individuals with diabetic peripheral neuropathy, while limited ankle dorsiflexion (diabetic equinus) has been reported, directly or indirectly, across multiple studies as a highly relevant pathomechanical characteristic.

The available evidence suggests that diabetic equinus may contribute to compensatory foot pronation, consistent with biomechanical mechanisms described in non‐diabetic populations. However, the literature remains fragmented and largely cross‐sectional, limiting the ability to establish causal relationships or validate progression models; therefore, no definitive etiological sequence can currently be verified.

Based on the data from this scoping review, future longitudinal studies are necessary to investigate temporal transitions between deformity stages, clarify the clinical implications of these biomechanical changes, and support the development of effective, targeted preventive strategies.

## Author Contributions

G.D.S., and R.C. are the project leaders and contributed to the study's methodological aspects, the development of the research team, and the progression of the protocol. R.C., and G.D.S. developed the protocol reporting and the current manuscript. V.S. an expert librarian at the Scientific Library of San Matteo Hospital, Pavia, Italy, will support the literature search strategy. A.D.S. a statistician, will support data extraction, develop the data extraction tables, and create the PRISMA flowchart. G.D.S., G.M., A.D.M., and R.C. formed the reviewer team and provided article analyses, contributing to data extraction and manuscript preparation. D.G.A., A.B. and F.C. with their methodological and clinical expertise, supervised the methodological quality assessment. In the case of disagreements, D.G.A., A.B. and F.C. intervened to facilitate resolution. G.D.S., G.M., R.C., D.G.A., and A.D.M. will write the final manuscript.

## Funding

The authors have nothing to report.

## Conflicts of Interest

The authors declare no conflicts of interest.

## Supporting information


Supporting Information S1

Supporting Information S2

Supporting Information S3

## Data Availability

The data that supports the findings of this study are available in the supplementary material of this article.
