# Supplementary material for: Biomechanical Evolution in the Neuropathic Diabetic Foot: A Scoping Review of Gait and Kinetic‐Kinematic‐Structural Adaptations in Diabetes
Source: Diabetes Metab Res Rev. 2026 Nov 4;42(8):e70248. doi: 10.1002/dmrr.70248 (PMC13732642; doi:10.1002/dmrr.70248)
Supplement: Supplementary file 1 [file DMRR-42-e70248-s001.docx]

**This file contains the research strings, dates, databases, results, and some notes.**

| **Date** | **Database** | **Search query** | **Search database** | **RESULTS** | **NOTES** |
| --- | --- | --- | --- | --- | --- |
| 04/12/2024 | **Pubmed** | ("Diabetic Foot"[MeSH] OR diabet* AND foot) AND ("Biomechanical Phenomena"[MeSH] OR biomechanic* OR gait OR (walk* AND strategy) OR (limb AND rotation) OR (pelvic AND misalignment) OR abduct* OR (ankle AND (equin* OR tight* OR dorsiflex*)) OR Windlass OR "flat foot" OR flatfoot OR (pes plano*) OR pronat* OR (medialization AND axis) OR (valgus AND (deformit* OR rearfoot)) OR (rearfoot AND eversion) OR "flexed knees") Filters: from 2014/1/1 - 2024/12/4 Sort by: Publication Date | Filters: from 2014/1/1 - 2024/12/4 Sort by: Publication Date | **700** |  |
| 04/12/2024 | **Embase** | ('diabetic foot'/exp OR 'diabetic foot' OR 'diabet*') AND ('foot'/exp OR 'foot') AND ('biomechanics'/exp OR 'biomechanics' OR 'biomechanic*' OR 'gait'/exp OR 'gait' OR ('walk*' AND ('strategy'/exp OR 'strategy')) OR (('limb'/exp OR 'limb') AND ('rotation'/exp OR 'rotation')) OR (('pelvic'/exp OR 'pelvic') AND 'misalignment') OR 'abduct*' OR (('ankle'/exp OR 'ankle') AND ('equin*' OR 'tight*' OR 'dorsiflex*')) OR 'windlass' OR 'flat foot'/exp OR 'flat foot' OR 'flatfoot'/exp OR 'flatfoot' OR 'pes plano*' OR 'pronat*' OR (('medialization'/exp OR 'medialization') AND ('axis'/exp OR 'axis')) OR ('valgus' AND ('deformit*' OR 'rearfoot'/exp OR 'rearfoot')) OR (('rearfoot'/exp OR 'rearfoot') AND ('eversion'/exp OR 'eversion')) OR 'flexed knees') AND [01-01-2014]/sd NOT [05-02-2024]/sd | Filters:AND ('article'/it OR 'article in press'/it OR 'editorial'/it OR 'letter'/it OR 'preprint'/it OR 'review'/it OR 'short survey'/it) AND ('article'/it OR 'article in press'/it OR 'editorial'/it OR 'letter'/it OR 'preprint'/it OR 'review'/it OR 'short survey'/it) | **900** |  |
| 05/12/2023 | **Cochrane** | ("Diabetic Foot"[MeSH] OR diabet* AND foot) AND ("Biomechanical Phenomena"[MeSH] OR biomechanic* OR gait OR (walk* AND strategy) OR (limb AND rotation) OR (pelvic AND misalignment) OR abduct* OR (ankle AND (equin* OR tight* OR dorsiflex*)) OR Windlass OR "flat foot" OR flatfoot OR (pes plano*) OR pronat* OR (medialization AND axis) OR (valgus AND (deformit* OR rearfoot)) OR (rearfoot AND eversion) OR "flexed knees") | Splittato su più linee con termini mesh e uniti con # 86 Embase 63 PubMed 10 Cinahl | **98** |  |
| 05/12/2024 | **Pedro** | Abstract and title: "Diabetic Foot" Body part: foot or ankle | New records added since: 01/01/2024 | **57** | 3 Chinese language articles removed  (No english abstract) |
| 05/12/2024 | **Cinahl** | ((MH "Diabetic Foot") OR diabet* AND foot) AND ((MH "Biochemical Phenomena") OR biomechanic* OR gait OR (walk* AND strategy) OR (limb AND rotation) OR (pelvic AND misalignment) OR abduct* OR (ankle AND (equin* OR tight* OR dorsiflex*)) OR Windlass OR "flat foot" OR flatfoot OR (pes plano*) OR pronat* OR (medialization AND axis) OR (valgus AND (deformit* OR rearfoot)) OR (rearfoot AND eversion) OR "flexed knees") | Publication Date: 20140101-20241231 Academic journal | **289** |  |
|  |  |  | Total abstract from various databes | **2044** |  |
|  |  |  | Duplicates | **781** |  |
|  |  |  | Total articles | **1263** |  |
|  |  |  |  |  |  |

**Supplemental material. Appendix 1.**

**Appendix 2. Update December 2024- April 2026.**

| **Date** | **Database** | **Search query** | **search database** | **NOTE** | **RESULTS** |
| --- | --- | --- | --- | --- | --- |
| **03/05/2026** | **Pubmed** | **("Diabetic Foot"[MeSH] OR diabet* AND foot) AND ("Biomechanical Phenomena"[MeSH] OR biomechanic* OR gait OR (walk* AND strategy) OR (limb AND rotation) OR (pelvic AND misalignment) OR abduct* OR (ankle AND (equin* OR tight* OR dorsiflex*)) OR Windlass OR "flat foot" OR flatfoot OR (pes plano*) OR pronat* OR (medialization AND axis) OR (valgus AND (deformit* OR rearfoot)) OR (rearfoot AND eversion) OR "flexed knees") Filters from 2024/12/5 - 2026/4/30 Sort by: Publication Date** | **Filters: from 2024/12/5 - 2026/4/30 Sort by: Publication Date** |  | **168** |
| **03/05/2026** | **Embase** | **('diabetic foot'/exp OR 'diabetic foot' OR 'diabet*') AND ('foot'/exp OR 'foot') AND ('biomechanics'/exp OR 'biomechanics' OR 'biomechanic*' OR 'gait'/exp OR 'gait' OR ('walk*' AND ('strategy'/exp OR 'strategy')) OR (('limb'/exp OR 'limb') AND ('rotation'/exp OR 'rotation')) OR (('pelvic'/exp OR 'pelvic') AND 'misalignment') OR 'abduct*' OR (('ankle'/exp OR 'ankle') AND ('equin*' OR 'tight*' OR 'dorsiflex*')) OR 'windlass' OR 'flat foot'/exp OR 'flat foot' OR 'flatfoot'/exp OR 'flatfoot' OR 'pes plano*' OR 'pronat*' OR (('medialization'/exp OR 'medialization') AND ('axis'/exp OR 'axis')) OR ('valgus' AND ('deformit*' OR 'rearfoot'/exp OR 'rearfoot')) OR (('rearfoot'/exp OR 'rearfoot') AND ('eversion'/exp OR 'eversion')) OR 'flexed knees')AND [05-12-2024]/sd NOT [01-05-2026]/sd** | **Filters: AND ('article'/it OR 'article in press'/it OR 'editorial'/it OR 'letter'/it OR 'preprint'/it OR 'review'/it)** |  | **266** |
| **03/05/2026** | **Cochrane** | **("Diabetic Foot"[MeSH] OR diabet* AND foot) AND ("Biomechanical Phenomena"[MeSH] OR biomechanic* OR gait OR (walk* AND strategy) OR (limb AND rotation) OR (pelvic AND misalignment) OR abduct* OR (ankle AND (equin* OR tight* OR dorsiflex*)) OR Windlass OR "flat foot" OR flatfoot OR (pes plano*) OR pronat* OR (medialization AND axis) OR (valgus AND (deformit* OR rearfoot)) OR (rearfoot AND eversion) OR "flexed knees")** | Split across multiple lines using mesh terms and joined with # date range **05/12/2024 to 30/04/2026 2 Embase 6 PubMed  1 Embase e PubMed** |  | **9** |
| **03/05/2026** | **Pedro** | **Abstract and title: "Diabetic Foot" Body part: foot or ankle** | **New records added since: 06/12/2024** |  | **7** |
| **03/05/2026** | **Cinahl** | **((MH "Diabetic Foot") OR diabet* AND foot) AND ((MH "Biochemical Phenomena") OR biomechanic* OR gait OR (walk* AND strategy) OR (limb AND rotation) OR (pelvic AND misalignment) OR abduct* OR (ankle AND (equin* OR tight* OR dorsiflex*)) OR Windlass OR "flat foot" OR flatfoot OR (pes plano*) OR pronat* OR (medialization AND axis) OR (valgus AND (deformit* OR rearfoot)) OR (rearfoot AND eversion) OR "flexed knees")** | **Publication Date: 12/2024 - 04/2026 Academic article** |  | **51** |
|  |  |  |  | **Total articles** | **501** |
|  |  |  |  | **Duplicates from Refworks** | **165** |
|  |  |  |  | **Total articles after duplicates removed** | **336** |
|  |  |  |  |  |  |
|  |  |  |  |  |  |
|  |  |  |  |  |  |
|  |  |  |  |  |  |
