# Supplementary material for: Biomechanical Evolution in the Neuropathic Diabetic Foot: A Scoping Review of Gait and Kinetic‐Kinematic‐Structural Adaptations in Diabetes
Source: Diabetes Metab Res Rev. 2026 Nov 4;42(8):e70248. doi: 10.1002/dmrr.70248 (PMC13732642; doi:10.1002/dmrr.70248)
Supplement: Supplementary file 2 [file DMRR-42-e70248-s002.docx]

**The file contains the data extraction table with the paper’s AUTHOR(s), DOI, YEAR of publication, TYPE OF STUDY, POPULATION, CONTEXT, CONCEPT, RESULTS, CLINICAL RELEVANCE, and some NOTES.**

| AUTHOR | DOI | YEAR | TYPE OF STUDY | POPULATION | CONTEXT | CONCEPT | RESULTS AND CLINICAL RELEVANCE | NOTE |
| --- | --- | --- | --- | --- | --- | --- | --- | --- |
| Kahveci, A. et al. | 10.1016/j.foot.2024.102097 | 2024 | Cross-sectional controlled study | n=28; DPN n=14 vs. healthy controls n=14; 18–65 years | Laboratory (Türkiye); Functional Reach Test (FRT) | Dynamic postural control and neuromuscular strategies (CoM–CoP) | DPN alters balance strategies by integrating segmental kinematics and neuromuscular signals | Instruments: force plate, Xsens MVN, sEMG. Limited by small sample and lack of trunk sEMG. |
| Yamasaki, H. et al. | 10.1007/s13340-023-00647-9 | 2023 | Cross-sectional study | n=42; DM n=20, DPN n=15, DFU n=11; T1DM/T2DM | Laboratory (Japan); 2D gait analysis and standing balance | Progression of distal joint limitations and CoM instability | Distal joint limitations and CoM instability emerge in the DFU phase with compensatory gait adaptations | Tools: 2D video, Wii Balance Board. Lacks plantar pressure/kinetics; cross-sectional design. |
| Martin, S. et al. | 10.1186/s12938-023-01082-2 | 2023 | Cross-sectional controlled experimental study | n=43; Healthy young (16), Healthy elderly (14), Diabetes elderly (13) | Laboratory (Australia); treadmill with visual biofeedback | Gait adaptation (step length and toe clearance) as fall risk | Older adults with DM show inability to adapt gait to dynamic stimuli, increasing trip risk | Tools: Vicon, MATLAB. Lacks multi-segment kinetics; sample excluded neuropathy. |
| Zhang, L.-Y. et al. | 10.3390/ijerph20043688 | 2023 | Observational experimental study | n=19; women with early DM; 57–75 years | Laboratory (Hong Kong); barefoot walking at 3 speeds | Effect of speed on dynamic foot deformation and plantar pressure | Provides biomechanical basis for offloading footwear by mapping speed/loading interaction | Tools: 4D foot scanner, Pedar. Limited to women; small sample; lacks joint kinetics. |
| Saroha, A. et al. | 10.4103/jmu.jmu_109_22 | 2023 | Controlled cross-sectional study | n=110; T2DM n=55 vs. healthy controls n=55 | Outpatient (India); static ultrasound assessments | Morphological alterations of Achilles tendon (AT) and plantar fascia (PF) | Thickening and softening of AT/PF in DM may compromise load absorption/transmission | Tools: B-mode ultrasound, SWE. No neuropathy stratification or dynamic measurements. |
| Bartolo, E. et al. | 10.1016/j.gaitpost.2022.12.018 | 2023 | Cross-sectional study | n=80; DM, DPN, DNU, DHNU (20 each); T2DM | Laboratory (Malta); barefoot walking on pressure platform | Role of PTI vs. MPPP in ulcer risk | PTI discriminates presence of active/previous ulceration better than MPPP (hallux) | Instruments: Tekscan HR Mat. Site-specific analysis; PAD strictly excluded. |
| Deschamps, K. et al. | 10.1016/j.clinbiomech.2022.105802 | 2022 | Cross-sectional study | n=39; DPN n=13, DM n=13, healthy n=13; T1DM/T2DM | Multi-center laboratory (Belgium); speed-controlled barefoot walking | Kinematics and joint kinetics of the foot | Identifies "energy-neutral foot" via loss of midfoot power generation, driving joint stress | IOR-4segment model. Lacks EMG and direct tissue stiffness measurements. |
| Tramonti, C. et al. | 10.1016/j.clinbiomech.2022.105759 | 2020 | Cross-sectional study | n=100; Obese DM/DPN, Obese, Non-obese DM/DPN, Healthy (25 each) | Laboratory (Italy); 3D barefoot gait analysis | Synergistic effects of DM and obesity on walking | Combined DM and obesity lead to conservative gait with distal propulsive deficits and instability | Tools: ELITE system, Davis protocol. Lacks joint kinetics and plantar pressure. |
| Cao, Z. et al. | 10.1155/2022/2437831 | 2022 | Cross-sectional study | n=112; DPN+PAD, DPN, PAD, DM, Healthy | Laboratory (China); shod walking at self-selected speed | Effects of DPN and PAD on plantar pressure and COP displacement | DPN increases forefoot load; DPN+PAD increases high-load area and reduces midfoot support | Instruments: T&T Medilogic in-shoe. Lacks joint kinematics; shod gait only. |
| Jeong, H.-J. et al. | 10.1016/j.clinbiomech.2022.105662 | 2022 | Cross-sectional observational study | n=107; DMPN n=60; elderly n=22; young n=25 | Laboratory (USA); barefoot single-limb heel rise on force plate | Inter-segmental coordination between midfoot and ankle | DPN involves a loss of coordination that contributes to deformity progression and pathological loads | Focuses on heel-rise task; lacks kinetic and EMG measures during gait. |
| Allan, D. et al. | 10.1016/j.diabres.2022.109865 | 2022 | Cross-sectional observational study | n=39; T1DM/T2DM with bilateral neuropathy | Laboratory (India); barefoot walking and tissue hardness tests | Relationship between load exposure (PTI) and tissue biomechanics | Prolonged weight bearing (PTI) is associated with reduced plantar tissue hardness (heel/1st MTH) | Tools: Shore-00 hardness, Tekscan. Lacks skin thickness measurement. |
| Mustapa, A. et al. | 10.1155/2016/9305025 | 2016 | Systematic review (PRISMA) | Review of 38 studies (n=883 subjects with DPN) | Systematic review of posture and gait literature | Postural alterations and gait performance in DPN and fall risk | Consolidates evidence that DPN causes postural instability and conservative gait related to falls | Heterogeneous tools and settings; primarily cross-sectional source studies. |
| Chuter, V.H. et al. | 10.1186/s13047-021-00494-4 | 2021 | Cross-sectional study | n=136; T1DM/T2DM; 31.6% with neuropathy | Clinical (Australia); clinical foot evaluation and walking | Clinical measures (ROM/deformity) to identify high plantar pressure | Having ≥2 foot pathologies identifies subjects with pathological loading; useful for screening | Measurements: Ankle DF, Hallux ROM, HAV. Lacks joint kinetics. |
| Chatrenet, A. et al. | 10.1111/dme.14572 | 2021 | Controlled cross-sectional pilot study | n=42 DM (various PN levels) + healthy controls n=12 | Laboratory (France); Zebris FDM-S pedobarographic platform | Spatial patterns of plantar pressure using SPM | SPM identifies specific load patterns across neuropathy stages undetectable by regional analysis | Innovative methodology. Limited by small sample and cross-sectional design. |
| Menzildzic, S. et al. | 10.1016/j.foot.2021.101810 | 2021 | Cross-sectional study | n=33 diabetic feet without neuropathy (various HV stages) | Primary care/Laboratory (Canada); barefoot walking | Hallux Valgus (HV) stages and plantar pressure distribution | Severe HV leads to critical pressure increases at M1, justifying high-risk categorization | Lacks PTI and kinematics; exclusion of neuropathy limits generalizability. |
| Zellers, J.A. et al. | 10.1016/j.clinbiomech.2021.105371 | 2021 | Cross-sectional study | n=60; T2DM with peripheral neuropathy | Laboratory (USA); barefoot AROM, sit-to-stand, and walking | Toe extension magnitude/variability in relation to MTP2 deformity | High-magnitude, low-variability toe extension explains 47% of MTP2 deformity variance | Mechanisms: MRI-derived muscle ratio, MNSI. Lacks prospective outcomes. |
| Sadler, S.G. et al. | 10.1016/j.clinbiomech.2021.105324 | 2021 | Cross-sectional study | n=80; Elderly ≥65 years; DM n=40 vs. controls n=40 | Clinical/Laboratory (Australia); barefoot walking and lunge test | Effect of weight-bearing ankle equinus on forefoot loading | Ankle equinus is a mechanical determinant of forefoot loading independent of diabetes | Tools: Tekscan, Lunge test. Barefoot only; lacks kinematics/kinetics. |
| Jeong, H.-J. et al. | 10.1016/j.gaitpost.2020.11.013 | 2021 | Cross-sectional study | n=60 DMPN vs. non-DMPN reference group n=47 | Laboratory (USA); 3D kinematics of heel rise and walking | Midfoot and ankle kinematics during push-off | Identifies "midfoot collapse" subgroup with persistent dorsiflexion and high ulcer risk | Heel-rise performance reflects gait mechanics; lacks prospective ulcer data. |
| Chicharro-Luna, E. et al. | 10.1016/j.jtv.2021.04.001 | 2021 | Cross-sectional study | n=111; DM1/DM2 with and without DPN | Clinical (Spain); podiatric clinical evaluation | Relationship between DPN and prevalence of foot deformities | DPN is selectively associated with central digital deformities and hallux extensus | No functional or load analysis; unbalanced sample. |
| Korada, H.Y. et al. | 10.14341/DM12723 | 2021 | Cross-sectional study | n=60 T2DM; DNN, DPN, DNPAD groups (20 each) | Clinical (India); walking on WinTrack platform | Loading parameters across neuropathy and PAD groups | DNPAD combination creates the most unfavorable loading profile (↑MPP, ↑PTI, ↓area) | Supports the "neuroischemic foot" as a distinct biomechanical entity. |
| Amemiya, A. et al. | 10.3390/s20174863 | 2020 | Case-control study | n=59 pts with diabetic neuropathy; mean age ~66 yrs | Clinical/Laboratory (Japan) | Gait and footwear factors associated with shear stress | Specific motor patterns (↓knee flexion) and poor shoe fit are linked to site-specific calluses | Short-distance walking; mild-moderate neuropathy sample. |
| Gnanasundaram, S. et al. | 10.1016/j.fas.2019.01.005 | 2020 | Cross-sectional study | n=53; DM, DN, Healthy controls | Clinical (India) | Early identification of gait and pressure alterations | COP changes and increased heel pressure appear before structural foot deformities | Limited by sample size; barefoot only; lacks direct kinematics. |
| Henderson, A.D. et al. | 10.1155/2019/6985148 | 2019 | Case-control study | n=28; DPN n=14 vs. healthy controls n=14; ~61 yrs | Laboratory (USA); speed-controlled walking (1.0 m/s) | Kinematic and kinetic gait compensations in DPN | Neuropathic gait involves mechanical work shift from distal to proximal (crouch gait pattern) | Small sample; early DPN participants. |
| Abri, H. et al. | 10.1007/s40200-019-00387-1 | 2019 | Cross-sectional study | n=142 T2DM; (Absent, Mild, Mod, Severe neuropathy) | Clinical (Iran); static and dynamic barefoot assessment | Plantar pressure distribution across europathy severity | Increasing neuropathy severity is linked to ↑PP in the midfoot, heel, and medial forefoot | Confirms midfoot as a "sentinel" region for neuropathy progression. |
| Halawa, M. R. et al. | 10.1016/j.dsx.2017.09.010 | 2018 | Cross-sectional study | 50 adults with DM and 30 healthy controls | Outpatient | Plantar pressure and glycemic control | Provides regional loading data across the foot in relation to DM status | Descriptive focus on specific metatarsal and heel regions. |
| Searle, A. et al. | 10.1186/s13047-018-0306-x | 2018 | Cross-sectional study | 104 adults with DM and 40 without | Outpatient | Equinus and clinical impact on forefoot pressure | Focuses on equinus-related hyperload; suggests temporal contact duration as a factor | Does not account for abnormal pronation or first ray hypermobility. |
| Scarton, A. et al. | 10.1016/j.gaitpost.2017.07.117 | 2017 | Case -control study | 10 diabetic and 10 healthy adults | Outpatient | Lower-limb joint kinematics and kinetics | Changes observed in muscle strength and ankle stiffness during gait | Limited by very small sample size. |
| Petrovic, M. et al. | 10.1016/j.gaitpost.2017.05.016 | 2017 | Case control study | 14 DPN, 22 non-DPN DM, 31 healthy adults | Outpatient clinic | Joint moment arms and mechanical advantage | DPN reduce ankle moments and force requirements, but lose elastic energy efficiency | Confirms "hip strategy" compensations to offset distal deficits. |
| Searle, A. et al. | 10.1016/j.clinbiomech.2017.01.021 | 2017 | Systematic review and meta-analysis | Adults with DM with and without neuropathy | Outpatient clinic | Ankle DF ROM and plantar pressure | Confirms association between neuropathy, relative equinus, and increased hyperload | Notes issues with measurement standardization (e.g., Lunge Test). |
| Hazari, A. et al. | 10.1186/s40064-016-3405-9 | 2016 | Systematic review and meta-analysis | Adults: DM, DPN, and healthy controls | Outpatient | Spatiotemporal, kinematic, and kinetic gait parameters | Validates classification system based on plantar pressures for ulcer risk stratification | Robust for risk assessment but lacks specific joint pathomechanics data. |
| Allan, J. et al. | 10.1177/0309364615592705 | 2015 | Narrative literature review | Adults with Diabetes | Outpatient | Development of neuropathic foot deformities | Muscle weakness and limited ROM are primary factors in deformity and ulcer risk | General overview; identifies cross-sectional limitations in the field. |
| Molines-Barroso, R. J. et al. | 10.1016/j.diabres.2016.01.008 | 2016 | Cross-sectional study | DM with history of forefoot ulcers | Outpatient (Secondary prevention) | Risk stratification based on foot morphology | Pronated foot posture (FPI-6) is a significant risk factor for re-ulceration | Recommends FPI as a routine clinical screening tool. |
| Hastings, M. K. et al. | 10.1016/j.clinbiomech.2015.11.001 | 2016 | Cross-sectional study | 23 DM/DPN and 12 healthy | Outpatient | Midfoot alignment and column supporting structures | Deterioration of intrinsic muscles and tendons predicts midfoot dysfunction | Does not directly assess dynamic work or fall risk outcomes. |
| Hazari, A. et al. | ResearchGate (302026000) | 2016 | Cross-sectional study | 20 DPN, 20 non-DPN DM, 20 healthy | Outpatient clinic | Kinematics, kinetics, and plantar pressure | Significant differences in gait cycle and pressure parameters across groups | Provides additional evidence for lower-limb gait alterations in DPN. |
| DiLiberto, F. E. et al. | 10.1016/j.jbiomech.2015.08.020 | 2015 | Case-control study | 15 DM/DPN and 15 healthy adults | Outpatient clinic | Power and work of the midfoot and hindfoot | DPN involves reduced power generation and excessive absorption, driving midfoot damage | Links neuropathy and abnormal pronation to increased risk factors. |
| DiLiberto, F. E. et al. | 10.1016/j.gaitpost.2015.07.012 | 2015 | Case-control study | 15 DM/DPN and 15 healthy adults | Outpatient clinic | Reduction of metatarsal motion | DPN pts without deformity exhibit reduced mobility across multiple metatarsals | Comprehensive kinematic profile of unified forefoot function. |
| Tang, U. H. et al. | 10.3402/dfa.v6.27593 | 2015 | Cross-sectional study | 74 participants with diabetes | Orthopedic Research Laboratory | Foot deformities associated with pressure distribution | Structural traits (HV, rigidus, pronation) are linked to increased loading | Relies on traditional mechanical interpretations; limited functional analysis. |
| Lu, Y. C. et al. | 10.1155/2015/326493 | 2015 | Comparative cross-sectional study | 6 DPN with 5th toe deformity vs. 6 controls | Outpatient clinic | Foot loading with 5th toe deformity | Increased load found at hallux and central forefoot in 5th toe deformity | Suggests support deficits drive deformities rather than deformities being primary. |
| Yang, C. et al. | 10.1111/wrr.12331 | 2015 | Observational study | 649 DM pts vs. 808 non-diabetics | Outpatient | Dynamic plantar pressure distribution during gait | DM pts exhibit increased peak force, pressure, and torque | Large sample size; highlights potential triggers for ulcer formation. |
| Hastings, M. K. et al. | 10.1016/j.clinbiomech.2014.08.011 | 2014 | Cross-sectional case-control study | 36 pts (Neuropathy + Medial column deformity, DM, Healthy) | Movement Analysis Laboratory | Gait spatiotemporal and pressure parameters | Inability to form a "rigid lever" during loading leads to midfoot joint and tissue injury | Focuses on abnormal force transmission through supporting structures. |
| Sacco, I. C. N. et al. | 10.1016/j.jbiomech.2014.04.007 | 2014 | Observational study | 21 DPN and 21 non-diabetic subjects | Biomechanical laboratory | Electromyographic (EMG) muscle activation patterns | Identifies shift in extensor strategy to compensate for ankle deficits during forward propulsion | Wavelet EMG analysis; lacks direct kinetic or mechanical power measures. |
| Pachioni, C. G. F. et al. | 10.1016/j.gaitpost.2014.05.063 | 2014 | Cross-sectional study | 57 low-risk DM and 57 controls | Diabetic foot clinic | Pressure distribution in relation to gait characteristics | Pathomechanics arise from movement alterations before structural deformities appear | Focuses on posture and early-stage DM; lacks proximal kinetic analysis. |
| Yavuz, M. et al. | 10.1016/j.clinbiomech.2013.11.003 | 2014 | Case-control study | 28 participants (DM with/without PN) and 11 healthy | Outpatient clinic | Plantar pressure and shear stress distribution | PTI and STI (shear) vary across foot regions and differ between DM groups | Methodology for DPN assessment is specific; barefoot gait analysis only. |
| Jane S.S.P. Ferreira et al. | [10.1016/j.gaitpost.2025.07.328](https://doi.org/10.1016/j.gaitpost.2025.07.328) | 2025 | Cross selectional study | 102 DPN and 42 healthy | Experimental/laboratory setting (Brazil); instrumental acquisitions quantifying the gait profile score (GPS) and gait variable scores (GVS) and their association with clinical outcomes, compared to a healthy control group. | Clinical outcomes were assessed using validated tools for NDP severity, symptoms, foot health, and overall quality of life. | This study reveals significant gait alterations in individuals with DPN through GPS, GVS, and MAP measures. Elevated GPS scores, comparable to those found in severe neuromuscular disorders, underscore the extensive motor alterations during walking. Hip rotation and flexion, pelvic tilt, and sagittal knee moment contributed most to overall gait deviations. Ankle dorsiflexion, commonly impaired in DPN, was also significantly affected. | This study reveals significant gait alterations in individuals with DPN through GPS, GVS, and MAP measures. Elevated GPS scores, comparable to those found in severe neuromuscular disorders, underscore the extensive motor alterations during walking. Hip rotation and flexion, pelvic tilt, and sagittal knee moment contributed most to overall gait deviations. Ankle dorsiflexion, commonly impaired in DPN, was also significantly affected. |
| Ashleigh Cheng et al | <https://doi.org/10.1016/j.jtv.2024.07.013> | 2014 | Cross selectional study | 122 diabetic adults | To investigate the contribution of foot type to plantar pressures in a community-dwelling adult population with diabetes. | Performing the FPI-6 along with other clinical measures: Foot Posture Index-6 (FPI-6) as a measure of foot type, barefoot plantar pressure (peak pressures and pressure-time integrals were measured), the presence of forefoot deformity, peripheral neuropathy, range of motion (ROM) of ankle dorsiflexion and first toe metatarsophalangeal joint (MTPJ), and demographic variables. | This study is not done by aware biomechanists | This study is not done by aware biomechanists |
| Ahmed Amarak et al | https://doi.org/10.3390/app16062956 | 2026 | Systematic survey | diabetes gait analysis with wearable sensors |  |  | This study adds nothing new | This study adds nothing new |
| Ronaldo H. Cruvinel-Júnior et al. | https://doi.org/10.1016/j.bjpt.2025.101213 | 2025 | RCT | 62 participants | Experimental setting | Foot and ankle exercises that target modifiable risk factors, such as peak pressure, ankle motion, and foot strength, may reduce ulcer risk and improve gait biomechanics, but the mechanisms underlying these improvements are unclear. | It is dedicated exclusively to ankle dorsiflexion | It is dedicated exclusively to ankle dorsiflexion |
| Noriaki Maeshige et al | 10.1177/15347346221148456 | 2025 | RCT | 13 participants | Experimental setting | Foot and ankle exercises that target modifiable risk factors, such as peak pressure, ankle motion, and foot strength, may reduce ulcer risk and improve gait biomechanics, but the mechanisms underlying these improvements are unclear. | It is dedicated exclusively to ankle dorsiflexion | It is dedicated exclusively to ankle dorsiflexion |
| Dylan Heino et al | https://doi.org/10.1016/j.jbiomech.2026.113201 | 2026 | Cross selectional study | 25 participants | Experimental setting | In-shoe and barefoot pressures were recorded during a 7.6-meter walk |  |  |
| Li WX, et al. | PMID: 27777208 | 2016 | Observational study | 303 participants (DM) | Diabetic foot clinic | Plantar pressure and shear stress distribution | Foot Load evaluation |  |
| AUTHOR | DOI | YEAR | TYPE OF STUDY | POPULATION | CONTEXT | CONCEPT | RESULTS AND CLINICAL RELEVANCE | NOTE |
