# Supplementary material for: Biomechanical Evolution in the Neuropathic Diabetic Foot: A Scoping Review of Gait and Kinetic‐Kinematic‐Structural Adaptations in Diabetes
Source: Diabetes Metab Res Rev. 2026 Nov 4;42(8):e70248. doi: 10.1002/dmrr.70248 (PMC13732642; doi:10.1002/dmrr.70248)
Supplement: Supplementary file 3 [file DMRR-42-e70248-s003.docx]

| **Granularity Level** | **Description** | **Examples** |
| --- | --- | --- |
| High | Highly detailed, quantitative, continuous data | Plantar pressure maps (pixel-level or sensor-level); Joint angles per frame (kinematic time series); Ground reaction forces |
| Medium | Aggregated or summarized quantitative data | Peak plantar pressure per region; Average joint angles per gait phase; Step length, cadence |
| Low | Qualitative or categorical descriptors | Gait pattern classification (e.g., normal vs. pathological); Presence/absence of asymmetry; Observational scoring |

**Levels of granularity: the file contains the papers' descriptions of granularity levels, along with examples illustrating the algorithm.**
